# Prenatal stress and subsequent exposure to chronic mild stress influence dendritic spine density and morphology in the rat medial prefrontal cortex

**DOI:** 10.1186/1471-2202-8-107

**Published:** 2007-12-19

**Authors:** Kimmo A Michelsen, Daniël LA van den Hove, Christoph Schmitz, Olivier Segers, Jos Prickaerts, Harry WM Steinbusch

**Affiliations:** 1 Department of Neuroscience, Faculty of Health, Medicine and Life Sciences, Maastricht University, P.O. Box 616, 6200 MD Maastricht, The Netherlands; 2 Department of Biology, Åbo Akademi University, Biocity, Tykistökatu 6 A, 20520, Turku, Finland; 3 European Graduate School of Neuroscience (EURON)

## Abstract

**Background:**

Both prenatal stress (PS) and postnatal chronic mild stress (CMS) are associated with behavioral and mood disturbances in humans and rodents. The aim of this study was to reveal putative PS- and/or CMS-related changes in basal spine morphology and density of pyramidal neurons in the rat medial prefrontal cortex (mPFC).

**Results:**

We show that rats exposed to PS and/or CMS display changes in the morphology and number of basal spines on pyramidal neurons in the mPFC. CMS had a negative effect on spine densities, particularly on spines of the mushroom type, which are considered to form stronger and more stable synapses than other spine types. PS alone did not affect spine densities, but had a negative effect on the ratio of mushroom spines. In addition, PS seemed to make rats less responsive to some of the negative effects of CMS, which supports the notion that PS represents a predictive adaptive response.

**Conclusion:**

The observed changes may represent a morphological basis of PS- and CMS-related disturbances, and future studies in the field should not only consider total spine densities, but also separate between different spine types.

## Background

An increasing amount of evidence indicates that exposure to prenatal stress (PS) increases the risk for developing psychopathology later in life [1]. In humans, PS has been associated with learning, behavioral and mood disorders, and rodent studies have linked PS to increased anxiogenic and depressive-like behavior and morphological and neurochemical changes in the brain [2,3]. Recent additions to the growing evidence include the demonstration of profound changes in dendritic arborization and spine densities in the rodent brain [4,5]. Exposure to chronic mild stress (CMS) in adulthood has also been linked to behavioral disturbances [6], as well as altered dendritic morphology [7].

The predictive adaptive response hypothesis states that the fetus responds to cues, which might be predictive of its future environment, and adapts its physiology accordingly. Thus, PS-related changes may provide a survival advantage if the offspring is born into a stressful environment. Some studies suggest that this may indeed be the case [8].

**Figure 1:**
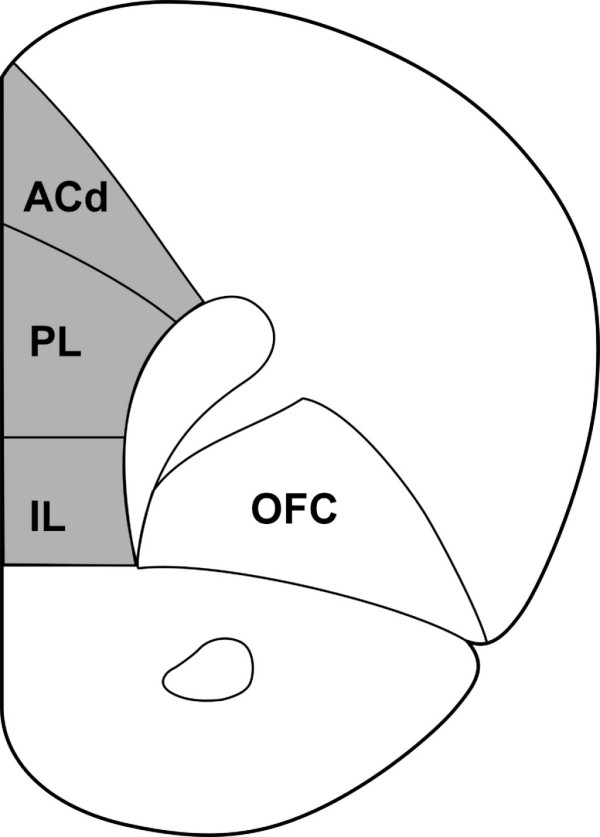
A coronal section through the rat brain illustrating the mPFC (shaded area). The mPFC consists of a dorsal mPFC (dorsal anterior cingulate cortex and dorsal part of the prelimbic cortex) and a ventral mPFC (ventral part of the prelimbic cortex and infralimbic cortex) [9]. Neurons were filled in coronal sections at approximately 1.7 mm to 3.7 mm from bregma. ACd: dorsal anterior cingulated cortex; PL: prelimbic cortex; IL: infralimbic cortex; OFC: orbitofrontal cortex. Modified after [35].

Of special interest in the psychopathology of PS and CMS is the medial prefrontal cortex (mPFC). The rat mPFC consists of a dorsal part, which includes the anterior cingulate cortex, and a ventral part [9] (Fig. 1). Both parts are implicated in executive function and, thus, the mPFC provides flexibility to affective processing [10,11]. Moreover, it has recently been demonstrated that both the dorsal [12,13] and ventral part [14,15] of the mPFC determine how a stressor is controlled, at the level of brain structure activity as well as behavioral response. Thus, the mPFC may protect the subject against depression [16]. Along similar lines, the corresponding prefrontal areas in humans are decreased in volume [17], show a lower tissue organization [18,19] and have specific changes in energy metabolism [20] in depression.

In the present study we evaluated whether PS and/or CMS exposure later in life leads to morphological changes at a basic functional level, i.e. basal dendritic spine density and morphology, in layer II and III pyramidal neurons of the rat mPFC. The choice of layers was based on previous reports, which have shown stress-related changes in spine density in these layers [5,15]. For this purpose, we used a combination of intracellular iontophoretic injections with a fluorescent dye ("cell loading"), confocal microscopy and modern quantification software.

## Results

**Figure 2:**
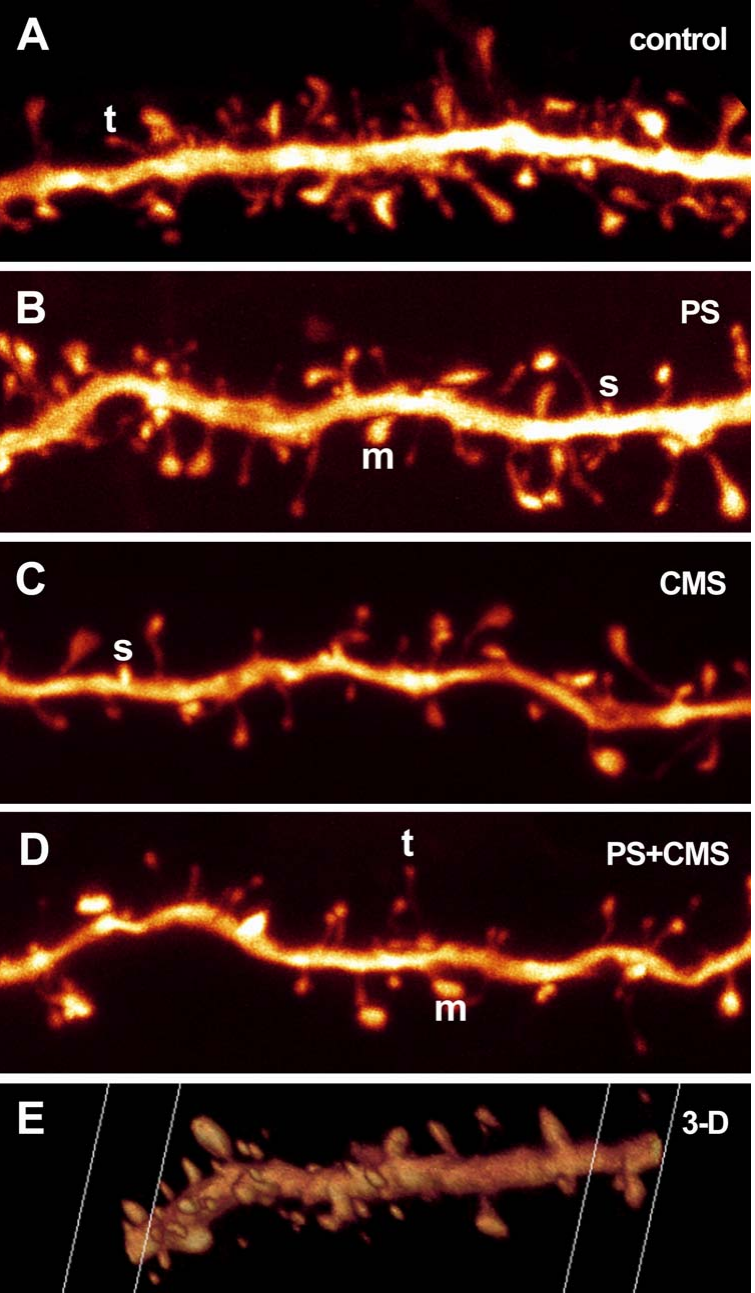
Maximum intensity projections of details from four of the stacks used for collection of spine data. The images are shown "raw" and have not undergone post-processing, such as contrast-enhancement (only rotation and resampling for screen-fit and printing purposes) (A-D). A screen shot of a three-dimensional (3-D) animation of one of the analyzed dendrites illustrates the 3-D advantage of the method (E). The letters t, m and s exemplify thin, mushroom and stubby spines, respectively.

**Figure 3:**
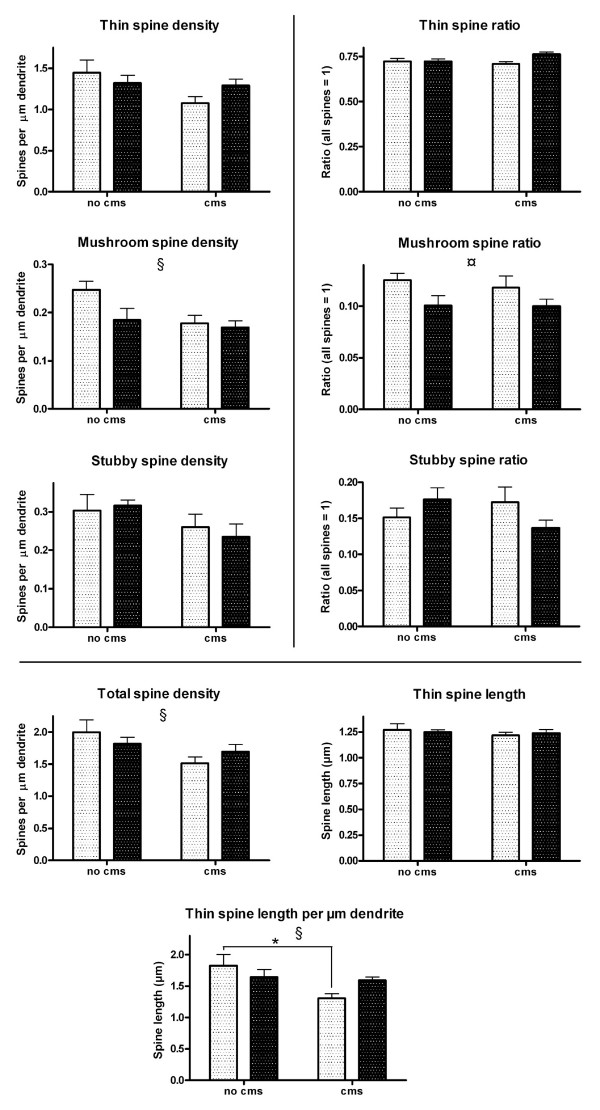
The densities (left column) and ratios (right column) of each spine type separately. The total density of spines, average length of thin spines and thin spine length per μm dendrite are shown at the bottom. Light columns = no PS; dark columns = PS. Error bars show SEM. § CMS effect (P < 0.05); ¤: PS effect (P < 0.05); *: P < 0.05 in Bonferroni post-hoc analysis. See also tables 1 and 2.

Quantitative confocal microscopy analysis of the dendritic spines, imaged with the help of an intracellularly injected fluorescent dye, revealed PS- and/or CMS-related changes in spine number and/or morphology (Fig. 2). The measured parameters were: 1) Spine density, i.e. spine number per μm dendrite, which was expressed separately for each spine type (thin, mushroom, stubby) and for all types together (total spine density); 2) spine ratio, i.e. the abundance of each spine type relative to the total spine density; 3) the average thin spine length; 4) a combination of thin spine density and average thin spine length, i.e. thin spine length per μm dendrite. The purpose of this combined measure was to detect putative subtle thin spine dynamics, such as shrinkage, complete degeneration, or transformation to another spine type, which might go unnoticed when considering measures 2 and 3 separately. The results of the statistical analysis of spine type densities and ratios are presented in Table 1 and Fig. 3, and of total spine density, thin spine length and thin spine length per μm dendrite in Table 2 and Fig. 3.

**Table 1:** Statistical analysis

	Spine type:	Thin		Mushroom		Stubby	
		F value	P value	F value	P value	F value	P value

**Spine density**	PS effect	0.17	0.686	3.67	0.079	0.04	0.848
	CMS effect	3.56	0.084	5.33	**0.040**	3.63	0.081
	PSxCMS interaction	2.56	0.136	2.16	0.167	0.36	0.562
							
**Spine ratio**	PS effect	3.52	0.085	5.92	**0.032**	0.13	0.730
	CMS effect	0.89	0.365	0.22	0.647	0.34	0.573
	PSxCMS interaction	3.65	0.080	0.15	0.707	3.79	0.075

Results of statistical analysis (F and P values) with generalized linear model MANOVA for each spine type separately.

**Table 2:** Statistical analysis

	Total spine density		Thin spine length		Thin spine length/ μm dendrite	
	F value	P value	F value	P value	F value	P value

PS effect	0.00	0.988	0.00	0.965	0.23	0.644
CMS effect	5.26	**0.041**	0.67	0.431	6.03	**0.030**
PSxCMS interaction	1.73	0.207	0.32	0.584	3.89	0.072

Results of statistical analysis (F and P values) with ANOVA for the total density of spines, average length of thin spines and thin spine length per μm dendrite.

CMS had a clear negative effect on total spine density (Table 2 and Fig. 3) and on the density of spines of the mushroom type (Table 1 and Fig. 3), but not on the density of the two other spine types (Table 1 and Fig. 3). CMS did not have an effect on the average length of thin spines (Table 2 and Fig. 3). However, when average thin spine length and density were considered together, expressed as thin spine length per μm dendrite, CMS had a negative effect (Table 2 and Fig. 3). PS had a clear effect only on the ratio of mushroom spines (Table 1 and Fig. 3).

## Discussion

CMS had a negative effect on the basal spine density of layer II and III pyramidal neurons in the left hemisphere of rat mPFC. This indicates that spines and, hence, probably also synaptic contacts are lost due to CMS. Spines of the mushroom type, characterized by a large spine head, were particularly affected. Spine head size correlates with post-synaptic density area and the number of presynaptic vesicles and has thus been suggested to reflect differences in synaptic efficacy [21]. Furthermore, large spines are more likely than small spines to contain smooth endoplasmic reticulum [22], which indicates differences in calcium-handling, as suggested [23]. In addition, mushroom spines seem to form more stable synapses than thin spines, which have higher motility and form more transient contacts [24]. Thus, it can be speculated that the loss of mushroom spines has a more profound effect on neuron function than the loss of the other types does, and than what could be expected if only the loss of total spine numbers were considered. However, it must be kept in mind that some synapses may turn into shaft synapses, as the spines are lost, and that shaft synapses cannot be quantified with the method we used.

Spines are dynamic and may change shape and size, as well as appear or disappear altogether [23]. In an attempt to evaluate whether spines were shrinking or growing due to PS and/or CMS, we measured the lengths of the thin spines (the other two types were not measured because due to their small or absent necks the results would have been too imprecise). Somewhat surprisingly, the average length of the spines was neither affected by CMS, nor PS nor by their combination. However, when the average length of thin spines was considered in combination with the density of thin spines (which showed a decreasing CMS-dependent trend) a negative CMS-effect was observed. This effect could possibly be attributed to putative conformational changes of the thin spines, as they are in the process of either transforming into another spine type or disappearing completely.

Our results show, that CMS leads to dendritic spine loss in the medial prefrontal cortex of the left hemisphere. Furthermore, PS seems to decrease the vulnerability to some of the degenerating effects of CMS. It is tempting to speculate that this is due to a predictive adaptive response, although it must be kept in mind that the current study does not provide convincing evidence for it. Nevertheless, it has been shown, that the fetal environment can influence the risk of postnatal disease and the ability to cope with the postnatal environment [25] and, indeed the difference in thin spine length per μm dendrite was significantly decreased in the CMS group as compared to controls, but not in the CMS+PS group, whose average (1.591) was close to that of the PS group (1.648). It should be noted, however, that the values for the CMS+PS group were not reversed to control levels, which could be expected of a strong predictive adaptive response. In addition, a trend towards an interaction between PS and CMS was observed with regard to spine length per μm dendrite and thin spine ratio and stubby spine ratio, which might reflect the observed putative compensatory effect of PS on CMS. The notion that PS could represent a predictive adaptive response, which makes rats less responsive, but not unresponsive, to the negative effects of CMS, is further supported by our observation that, when subjected to the home cage emergence test, CMS rats showed signs of increased anxiety. This effect was not observed in CMS rats, which had previously been exposed to PS (unpublished observation).

No effect of PS on spine density was observed. However, the negative effect of PS on the ratio of mushroom spines suggests that PS may induce some spine rearrangements in the neurons studied. The result becomes more interesting with regard to future studies when one considers the suggested relatively strong synaptic strength of mushroom spines, and the fact that PS showed a trend towards a decrease in mushroom spine density. Various studies have demonstrated a decrease in, for example, synaptophysin immunoreactivity, after PS [2,3]. In line with the fact that mushroom spines have a larger postsynaptic density and more synaptic vesicles than smaller spines do [21], quantification of immunoreactivity for postsynaptic density and synaptic vesicle markers in relation to synaptophysin immunoreactivity could further elucidate the dynamics of spines in the brain of rats exposed to PS.

In this study, we divided the spines into the three main categories introduced by Peters and Kaiserman-Abramof in 1970 [26]. However, in reality the various spine shapes fall along a continuum of different neck lengths and head sizes; even branched spines exist; see [23]. For example, Garcia-Lopez and coworkers used a classification into six types [27]. A more detailed analysis of spine morphology in combination with new software, which allows rapid and automated quantification of spine numbers and shapes, is likely to give new insights to spine dynamics in the near future; see [28] for some of the latest methodological advances.

A recent study by Murmu and coworkers [5] showed that PS correlates with changes in spine density and dendritic tree arborization in dorsal anterior cingulate (ACd) and orbitofrontal cortex (OFC). That study did not find a clear difference in total spine densities of the basal dendrites of ACd pyramidal neurons in male PS versus male control animals. The finding is in agreement with our study, which included the ACd in the mPFC area. Furthermore, Murmu and coworkers found that the apical dendrite spine density was decreased in male PS rats. We did not measure basal dendritic length, but they found no effect of PS on this measure. Along similar lines, several studies have reported that chronic stress in male rats only affects the length of apical dendrites but not of basal ones [7,29-31]. This suggests that it is feasible to assume that in our study basal dendrite arborization would not have been affected in any of the experimental groups. Further, chronic restraint stress has been shown to affect apical dendritic spine densities in the mPFC, whereas basal dendritic spines were not affected [32]. This is in contrast to our finding, which showed that spine densities on the basal tree were indeed affected. The discrepancy is probably due to the difference in stress (chronic restraint stress vs. variable CMS) and our inclusion of the infralimbic cortex.

Murmu and coworkers also studied the OFC after PS and found that spine densities in male rats were decreased both on apical and basal dendrites. In female rats, spine densities were decreased in both dendrite types in both ACd and OFC. In all cases of spine reductions in both females and males, this reduction was approximately 20% [5]. Yet, dendritic length was not affected in these brain areas of female rats after PS.

One can argue, that the behavioral testing of the rats used in this study may have affected spine morphology and numbers, but it has to be noted that rats were left undisturbed for two weeks after testing and testing itself did not include any chronic stressful events including administration of repeated food chocks or chronic restraint which is known to result in long-lasting structural changes in spines [33]. Furthermore, since all animals underwent exactly the same testing procedure, it is unlikely that the behavioral testing would be responsible for the significant effects of PS and/or CMS on spines presented here.

Due to limitations imposed by the fact that the rats, which were used for this experiment, were also used to study other, as yet unpublished, putative effects of PS and/or CMS, we decided to concentrate our efforts on basal spines only. For example, the animals had to be killed within a small time-window in order to exclude the possibility that different survival times after the behavioral experiments would affect the spine data. Cell loading is time-consuming and must be done within a matter of days after perfusion of the brain, so the time-restraint prevented us from loading enough neurons to be able to include both dendrite types in the analysis. With the current results at hand, it is evident that future experiments on the effects of PS and/or CMS on spine number and morphology could benefit from including both apical and basal dendrites. Studies involving the mPFC could also benefit from taking into account the heterogenicity of the mPFC, instead of treating it as one entity, in order to minimize bias introduced by possible differences in sampling within the chosen area, and to detect possible differences between the parts of the mPFC. In addition, they could benefit from analysing a larger number of animals per group than was done in this study, in order to provide more convincing statistical evidence of putative effects.

With these suggested improvements for future studies, we acknowledge the methodological limitations of the current one. Nevertheless, we present statistically significant results on the effects of PS and/or CMS on dendritic spines in the mPFC, which should encourage further, more detailed, studies on PS and/or CMS-related effects on the brain.

The method of cell loading (also called cell filling) in combination with laser confocal microscopy offers several clear advantages to, for instance, the traditional method of analyzing Golgi-preparations under an epifluorescence microscope: 1) Injecting a fluorescent dye into a single neuron makes it possible to analyze that neuron without interference from nearby dendrites. 2) Injection can be done at random, whereas it is not clear why the Golgi-method stains some neurons and leaves others unstained. 3) Laser confocal analysis of loaded dendrites and spines allows three-dimensional analysis, so that spines immediately below or above the dendrite can be distinguished. 4) The high resolution reveals spines, which go unnoticed in regular fluorescence microscopy, and allows one to distinguish different types of spines, as demonstrated here.

## Conclusion

In summary, this study is the first to show CMS-dependent morphological changes on the level of basal dendritic spines in the rat mPFC, and PS seems to make the brain less responsive to some of the stress-related changes as implicated by the predictive adaptive response hypothesis.

## Methods

### Animals

This experiment was approved by the Animal Ethics Board of the University of Maastricht, the Netherlands. Acclimatized pregnant Sprague-Dawley rats (Charles River, The Netherlands) were housed individually within a temperature-controlled environment (21 ± 1°C) with a 1:1 light:dark cycle (lights on at 7.00) and had access to food and water *ad libitum*.

Restraint stress was performed daily during the last week of pregnancy (embryonic day 14–21). Pregnant rats were restrained three times a day for 45 min in transparent plastic cylinders and simultaneously exposed to bright light, as described [34]. Female rats from the control group were left undisturbed in their home cage.

At postnatal day 21 (P21), male pups (n = 8/group) were weaned and housed together (2 rats/cage) and kept at a reversed day-night cycle from this point onward (lights on at 17.00) in order to make it practically feasible to perform behavioral experiments. At P77, 4 animals from each of the 2 groups were subjected to variable CMS for three weeks, resulting in the four following groups (n = 4/group): control (untreated), PS, CMS and PS+CMS. Stressors (housing in mice cage, cage tilt [angle of 45°], housing in an empty cage [no sawdust], wet bedding in cage [200 ml cold water added per cage], flashing light [stroboscope; low intensity, 2.5 Hz]) during the dark phase were applied in a random order. Two stressors per day, each lasting for three hours, were applied. Subsequently, the animals were subjected to various behavioral tasks, after which they were left undisturbed for approximately 2 weeks and used for the current study at P100.

### Tissue preparation

The rats were perfused transcardially with 1% paraformaldehyde for 1 min followed by a mixture of 4% paraformaldehyde and 0.125% glutaraldehyde in 0.1 M phosphate buffer, pH 7.4 (PB) for 11 min. The brain was removed and hemisected. The left hemisphere was postfixed for 4 h in the same mixture and then moved to PB with 0.02% NaN_3 _to await sectioning at 200 μm on a vibratome. The right hemisphere was used in another study.

### Cell loading

The sections were incubated with Hoechst (in PB) for at least 15 min to reveal the cell nuclei under UV illumination. They were then rinsed in PB, mounted on a nitrocellulose filter and immersed in PB under a fluorescence microscope (Leica DMLFS; Leica Microsystems, Heidelberg, Germany) equipped with a 40× water immersion objective. Using a glass micropipette attached to a micromanipulator (MP-85; Sutter instrument, Novato, CA, USA) a fluorescent dye (Lucifer Yellow CH lithium salt; Invitrogen, Carlsbad, CA, USA) was injected under a direct current of 1–6 nA for 6–12 min (current source: Model 260, World Precision Instruments, Sarasota, FL, USA) into layer II and III pyramidal neurons in the medial prefrontal cortex until the dye had reached the distal dendrites and no further loading was observed. The neurons were chosen in a systematic manner at approximately equal distances from each other, in an attempt to obtain an equal representation of subregions. The loaded neurons were sufficiently far apart for their dendritic trees not to overlap.

### Imaging

Three-dimensional image stacks of basal dendrites were collected with a Leica TCS-SP confocal laser scanning microscope system equipped with an Ar-Kr laser (Melles Griot, Carlsbad, CA, USA). LY was excited with the 488 nm laser line and emission was collected in the 490–690 nm interval. Confocal microscopy image stacks with an xy pixel size of 0.05 × 0.05 μm and a step-size of 0.2 μm between optical planes were acquired with a 100× n.a. 1.4 oil immersion objective with a theoretical lateral and axial resolution of 0.136 and 0.291 μm, respectively. Up to 3 stacks per neuron were collected at randomly selected sites at a radial distance of 25–105 μm from the soma. The average dendrite length per site was 33 μm, so the length per cell was up to 100 μm. Branch order was not addressed. The samples were first divided into proximal (25–55 μm from soma), medial (50–80 μm from soma) and distal (75–105 μm from soma) but were later merged because no significant differences could be seen between the three divisions.

### Spine quantification and measurements

The image stacks were opened in Neurolucida software (Microbrightfield, Williston, VT, USA) and spines were counted and labeled as thin, stubby or mushroom type based on morphology as follows: thin spines = long narrow necks and small to medium-sized heads; mushroom spines = short necks and big heads; stubby spines = short protrusion with no clear necks (Fig. 2). The length of the thin spines was measured with the same software. A total of 7426 spines were counted in 4–6 neurons/rat and 4 rats/group.

### Statistics

The effects of CMS and PS on the densities and ratios of thin, mushroom and stubby spines were evaluated using MANOVA. The effects on total spine density, average spine length and spine length per μm dendrite were evaluated using ANOVA (prenatal condition x postnatal condition) and analyzed in more detail using Bonferroni post-hoc tests. Statistical significance was defined as P < 0.05. All statistics were carried out using SPSS software version 14 (SPSS Inc, Chicago, IL, USA).

## Authors' contributions

KAM contributed to the design of the study, participated in the tissue preparation, performed the cell loading, confocal imaging, spine quantification and data collection, contributed to the statistical analysis and drafted the manuscript. DLAvdH contributed to the design of the study, performed the PS and CMS treatment and maintained the rats, contributed significantly to the statistical analysis and assisted substantially with drafting the manuscript. CS assisted with drafting the manuscript. OS assisted with the cell loading and performed the spine length measurements. JP contributed to the design of the study, contributed significantly to the PS and CMS treatment, maintenance of the rats and the statistical analysis and assisted substantially with drafting the manuscript. HWMS contributed to the design of the study and assisted with drafting the manuscript. All authors approved the manuscript.
